# PharmAI: domain-specific QA generation by adaptive learning and fine-tuning of small language models

**DOI:** 10.3389/frai.2026.1944175

**Published:** 2026-10-15

**Authors:** Shenbagarajan Anantharajan, Saranraj Loganathan, Rajkumaran Soundarraj, E Emerson Nithiyaraj

**Affiliations:** Mepco Schlenk Engineering College, Sivakasi, India

**Keywords:** FAISS (Facebook AI Similarity Search), LoRA (Low-Rank Adaptation), pharmaceutical QA, retrieval-augmented generation, small language models

## Abstract

**Background:**

Domain-specific medical question-answering (QA) models face bottlenecks due to scarce labeled data, specialized terminology, and constrained computational hardware.

**Objectives:**

The study aims to develop an efficient pharmaceutical question-answering (QA) system that can be trained and deployed on consumer-grade hardware using Small Language Models (SLMs).

**Materials and methods:**

The pipeline processes four pharmaceutical textbooks containing 3,977 total pages, producing 7,370 semantically similar text chunks and 2,144 synthetic QA pairs under four questioning categories: definition, mechanism, comparison, and clinical application. Three Small Language Models (SLMs), namely SmolLM2-1.7B-Instruct, Qwen2.5–1.5B-Instruct, and Llama-3.2–1B-Instruct, are fine-tuned under the same QLoRA (Quantized Low-Rank Adaptation) configurations and then evaluated with retrieval metrics (Recall@K, Mean Reciprocal Rank) and generation metrics (ROUGE-1/2/L, Semantic Similarity, BERTScore). Architectural decisions including NF4 4-bit quantization, CPU-based FAISS indexing, and completion-only loss masking are implemented due to hardware constraints and validated via ablation analysis.

**Results:**

SmolLM2-1.7B-Instruct, Qwen2.5–1.5B-Instruct, and Llama-3.2–1B-Instruct demonstrated identical retrieval performance, achieving a Recall@1 of 0.517, Recall@3 of 0.732, Recall@5 of 0.797, and an MRR of 0.628. Similarly, the generation performance evaluation revealed that Qwen2.5–1.5B FT outperformed the other fine-tuned SLMs, achieving the highest scores across all metrics (ROUGE-1 = 0.47, ROUGE-2 = 0.25, ROUGE-L = 0.38, and BERTScore = 0.72). In comparison, SmolLM2-1.7B FT and Llama-3.2–1B FT exhibited comparable performance, with ROUGE-1 scores of approximately 0.35, ROUGE-2 scores of 0.16–0.17, ROUGE-L scores of 0.25–0.26, and BERTScores of 0.66, indicating lower lexical and semantic alignment with the reference outputs than Qwen2.5–1.5B FT.

**Conclusion:**

We evaluate retrieval and generation performance using comprehensive metric suites, incorporating a category-wise breakdown that reveals comparison questions to be the most challenging for single-passage retrieval architectures.

## Introduction

The growing volume of pharmaceutical and biomedical literature has increased the need for QA systems that provide accurate, domain-specific information for both education and clinical practice in that domain. Though general Large Language Models (LLMs) show strong language understanding capabilities, they sometimes generate incorrect medical information, misinterpret drug mechanisms, or produce responses that are not based on pharmaceutical references. Retrieval-Augmented Generation (Lewis et al., 2020) helps overcome this limitation by incorporating relevant external knowledge during time of inference. The performance of a retrieval-augmented generation (RAG) system is determined not only by the quality of its retrieval mechanism but also by the language model’s capability to effectively leverage the retrieved contextual information during response generation. Another challenge while developing domain-specific pharmaceutical assistants is the limited availability of high-quality QA datasets that are created from standard pharmaceutical textbooks that are actually used by students in colleges. Existing biomedical datasets, like MedQA (Jin et al., 2021) and PubMedQA (Jin et al., 2019), primarily target medical examination-based questions and biomedical research literature. This limits the coverage of pharmaceutical concepts such as drug mechanisms, pharmacokinetics, dosage forms, and therapeutic applications. Constructing these datasets manually is time-consuming and needs heavy resources due to the huge variety and volume of data. This motivated the use of automated data generation techniques. Computational resources remain a practical constraint. Many biomedical LLMs require high-memory, multi-GPU systems for fine-tuning and deployment, limiting their accessibility for students and researchers with consumer-grade hardware. This research article proposes PharmAI, an end-to-end pipeline that automatically generates a pharmaceutical QA dataset from textbook content, fine-tunes Small Language Models using parameter-efficient techniques, integrates retrieval through a FAISS-based RAG pipeline, and evaluates using both retrieval and generation metrics. The entire workflow is designed and implemented on a single NVIDIA RTX 4050 Laptop GPU with 6 GB of VRAM.

Despite these advances, several key bottlenecks impede progress toward hardware-accessible pharmaceutical QA systems: (i) widely used biomedical benchmarks like MedQA and PubMedQA lack the granular pharmacokinetic and mechanism-of-action content typical of pharmaceutical textbooks; (ii) existing fine-tuned biomedical models rely on multi-GPU or TPU clusters inaccessible to resource-constrained researchers; (iii) naive fine-tuning on raw textbook text without completion-only loss masking causes small models to perform language continuation rather than targeted question answering; (iv) inconsistent publisher heading formats and fixed-length chunking obscure section hierarchies, diminishing retrieval precision; and (v) previous pharmaceutical RAG evaluations rarely benchmark multiple small language model architectures under uniform experimental conditions. The present study is designed to address each of these gaps directly.

This work is motivated by three primary factors: (i) the growing demand for accessible pharmaceutical educational resources; (ii) the lack of an open, end-to-end textbook-to-QA pipeline compatible with consumer-grade hardware; and (iii) the need for a rigorous, controlled comparison of small language models (SLMs) under identical computational conditions. The key contributions of this work, restated here as discrete points, are.

1. A page-aware PDF extraction and structure-preserving chunking pipeline that segments pharmaceutical textbooks into semantically coherent passages while respecting chapter and section boundaries across four distinct publisher heading conventions.
2. An automated synthetic QA generation framework using a LLM API, with five sequential quality filters that reject hallucinated or malformed pairs.
3. A hardware-constrained fine-tuning protocol employing QLoRA with NF4 4-bit quantization, completion-only loss masking, and a mixed-mode training strategy that prevents textbook-continuation artefacts observed in naive fine-tuning approaches.
4. A systematic comparative evaluation of three SLMs under identical configurations, using lexical (ROUGE) and semantic (BERTScore, cosine similarity) generation metrics alongside standard retrieval metrics, all executed within a 6 GB VRAM budget.

The remainder of this paper is organized as follows. The Literature Review section reviews related work in retrieval-augmented generation, parameter-efficient fine-tuning, and biomedical NLP and closes with a chronological comparison of prior studies. The Materials and Methods section describes the system architecture, including dataset preparation, chunking, QA generation, LoRA fine-tuning, the RAG inference pipeline, and the evaluation framework. The Experimental Setup section presents the hardware platform, dataset statistics, and evaluation protocol. The Results section reports retrieval and generation results, per-category analysis, statistical significance, and ablation findings. The Discussion section addresses corpus generalization, retrieval design choices, and quality-filtering trade-offs. The Conclusion and Limitations sections close the paper and outline future work.

## Literature review

### Retrieval-augmented generation

Lewis et al. (2020) introduced the retrieval-augmented generation (RAG) paradigm, coupling the parametric knowledge of a language model with non-parametric retrieval over a dense passage index. Dense passage retrieval using bi-encoder architectures (Karpukhin et al., 2020) demonstrated that the learned dense representations outperform sparse BM25 retrieval on open-domain QA benchmarks. Subsequent works have applied RAG to specialized domains including medicine [MedRAG] and law [LegalRAG], where up-to-date factual grounding is very critical. Authors employed a FAISS-based flat inner-product index (Johnson et al., 2021) with L2-normalised Sentence-Transformer embeddings (Reimers and Gurevych, 2019), which is mathematically equivalent to cosine similarity retrieval and well-suited to corpus sizes in the range of 5,000 to 20,000 passages without approximation error.

### Parameter-efficient fine-tuning

Hu et al. (2022) proposed Low-Rank Adaptation (LoRA), which approximates the weight update, with ∆W∈R ^ (d × k) as the product of two low-rank matrices B∈R ^ (d × r) and A∈R ^ (r × k), where r < min (d,k). As a result, the number of trainable parameters is reduced from (d × k) to r × (d + k) per each adapted layer. Similarly, Dettmers et al. (2023) demonstrated that NF4 Normal-Float quantization combined with double quantization of the scaling constants preserves perplexity within 1% of the full-precision baseline while also reducing model memory by nearly 75%. These techniques are central to the hardware feasibility of the proposed pipeline.

### Biomedical and pharmaceutical NLP (natural language processing)

Lee et al. (2020) and Gu et al. (2021) demonstrated that domain-adaptive pre-training on PubMed yields significant improvements on biomedical NER and relation extraction benchmarks. Specifically for pharma QA, Jin et al. (2021) introduced a dataset of US Medical Licensing Examination (USMLE) style questions, while Jin et al. (2019) constructed a biomedical yes/no/maybe QA dataset from PubMed abstracts. However, both corpora cover only general biomedical questions rather than the pharmacokinetic and mechanism-of-action characteristics of textbook contents, motivating the synthetic generation approach in the current work.

### Synthetic data generation for NLP

Instruction-following fine-tuning commonly employs model-generated synthetic data when human annotation is unavailable and expensive (Taori et al., 2023) [WizardLM]. Gilardi et al. (2023) showed that ChatGPT-generated annotations match or exceed crowd worker quality on several NLP benchmarks, supporting the viability of LLM-driven dataset construction. Quality filtering through overlap-based hallucination detection and format validation has been shown to improve the utility of synthetic training data (Wang et al., 2023). We applied five quality filters including a minimum 30% content-word overlap between generated answers and source passages, rejecting approximately 20% of raw API output generated. The literature reveals a clear research gap, as existing studies focus on general biomedical or clinical datasets and do not adequately address the challenges of generating high-quality, contextually grounded question-answer pairs from pharmaceutical textbooks while maintaining computational efficiency. Therefore, the present study proposes a comprehensive PharmAI framework that integrates page-aware document processing, retrieval-augmented generation, synthetic question-answer generation, and QLoRA-based fine-tuning of small language models to develop a domain-specific question-answering system capable of generating accurate, contextually grounded, and computationally efficient responses for pharmaceutical learning applications.

### Comparative summary of prior studies

Table 1 presents a chronological comparison of closely related studies, summarizing their methods, evaluation corpora, hardware requirements, and the specific limitations left unaddressed relative to our work.

**Table 1:** Comparative Summary of the related studies.

Year	Study	Method	Corpus	Hardware	Gap relative to this work
2020	Lewis et al.	DPR + seq2seq	NQ, TriviaQA	Multi-GPU	General domain; no pharmaceutical focus
2020	Karpukhin et al.	Bi-encoder retrieval	NQ, WebQ, TREC	Multi-GPU	Retrieval only; no generation or fine-tuning
2021	Geva et al.	FFN knowledge analysis	LAMA probe	Single GPU	Analysis only; motivates MLP-module targeting
2022	Hu et al.	Low-Rank adaptation	GLUE, NLG	Multi-GPU	No RAG; no biomedical domain
2023	Dettmers et al.	4-bit QLoRA	Vicuna benchmark	Single GPU	No RAG; no pharmaceutical evaluation
2023	Taori et al.	Synthetic instruct tuning	Self-generated	Single GPU	General domain; no structured QA-category generation
2024	Ben Allal et al.	SLM distillation	Curated web	Single GPU	No domain fine-tuning; no medical evaluation
2025	Present work	QLoRA + RAG pipeline	Synthetic, 4 pharma textbooks	RTX 4050, 6 GB	Addresses Gaps 1–5; full pipeline PDF to evaluation

### Objective of the research

This study aims to develop an automated pipeline for constructing domain-specific pharmaceutical AI assistants from textbooks, leveraging RAG and efficient fine-tuning techniques designed to run on consumer-grade hardware.

#### Research question

How can a retrieval-augmented, parameter-efficient small language model be developed to generate accurate and contextually grounded question-answer pairs from pharmaceutical textbooks?

## Materials and methods

### System design

The PharmAI pipeline comprises six stages, each consuming the output of the previous stage. Figure 1 illustrates the complete data flow from raw PDF input to evaluated RAG inference both in offline and online mode. Furthermore, Algorithm 1 shows QLoRA Fine-Tuning of Small Language Models for pharmaceutical QA.

**Figure 1:**
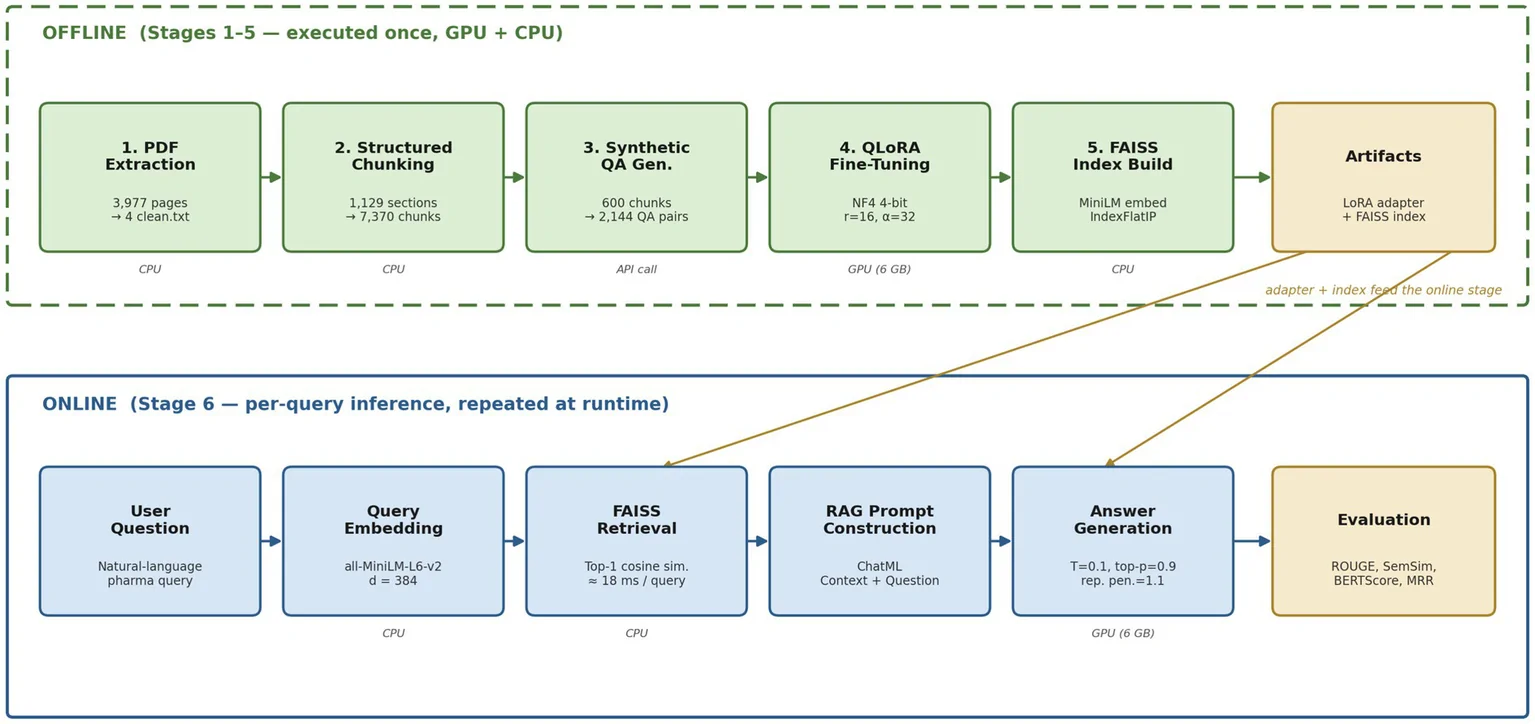
End-to-end architecture of the PharmAI pipeline. Dashed boundaries indicate offline stages; solid boundaries indicate online components.

#### Dataset preparation

The corpus consists of four pharmaceutical and biomedical science textbooks: a pharmacology textbook (533 pages), a pathology textbook (1,377 pages), a biochemistry textbook (672 pages), and a physiology textbook (1,395 pages), totaling 3,977 pages. All four volumes are standard graduate-level instructional texts covering topics directly relevant to pharmaceutical education, including drug absorption and distribution, mechanisms of action, adverse effects, pathological processes, and physiological systems. All training and evaluation data are derived from these four textbooks through the automated pipeline described in the upcoming subsections, eliminating dependence on pre-existing pharmaceutical QA corpora.

#### PDF processing and text extraction

Raw PDF files are processed using a page-aware extraction pipeline that produces two artifacts per volume: a cleaned plain text file with page-boundary markers in the format {PAGE N} and a structured record file (*.jsonl) containing one JSON record per page with fields {book, page, text}. The extraction procedure applies repeated line removal to eliminate publisher running headers and footers, which may contaminate the text index. Across the four volumes, 2,202 repeated lines were removed before downstream processing. Page markers are preserved throughout the pipeline to enable source attribution at the chunk level, allowing retrieval results to be mapped correctly to the respective textbook pages for re-verification.

#### Intelligent chunk generation

Effective retrieval requires text chunks that are semantically independent and sized to fit within the model’s effective context window. The chunking pipeline is done at two stages:

Stage C-1: *Chapter and Section Detection*: Four distinct heading conventions are observed across the four textbooks: chapter headings with spaces (C H A P T E R N) in pharmacology and pathology, sectional headings with dashed separation (SECTION-N) in physiology, and chapter headers in the form of ‘Chapter N: Title’ in biochemistry. A token level ‘ALL-CAPS heuristic’ matched at sentence boundaries detects sub-section headings within chapter content. Sentence boundaries are identified by the pattern [.!?]\s+[A−Z] . This prevents false splits on abbreviations. Each detected sectional heading produces a new record {book, chapter, chapter_num, section, page_start, text}. A stop-word set {CHAPTER, FIGURE, TABLE, …} prevents structural tokens from being wrongly classified as sectional headings. Across all four books, this procedure gave 1,129 section’s records.

Stage C-2: *Sentence-Boundary Chunking with Overlap*: Each section record is chunked as overlapping passages using a sliding-window. The target chunk length is 1,600 characters, nearly 400 tokens at the 4 character/token rate. An overlap of 320 characters is maintained between consecutive chunks to ensure that answer passages connecting chunks across boundaries remain accessible to the retriever. A max ceiling of 2,400 characters is enforced through word-boundary splitting to handle table content without sentence terminations. During post-processing, we merged tail chunks shorter than 120 characters into their predecessors and removed table-of-contents and index pages. The resulting corpus has 7,370 usable chunks, which are shown in Table 2.

**Table 2:** Per-volume chunking stats.

Volume	Pages	Sections	Chunks	Avg. chars	Max chars	TOC drop	Index drop
Pharmacology	533	204	899	1,421	2,397	0	13
Pathology	1,377	272	2,483	1,398	2,501	16	118
Biochemistry	672	204	912	1,387	2,415	17	3
Physiology	1,395	449	3,076	1,412	2,512	1	138
Total	3,977	1,129	7,370	1,404	-	34	272

#### Synthetic QA data generation

QA pairs are generated from Stage C-2 chunks using Google Generative AI (google-genai SDK). The used sampling procedure selects 600 chunks from the 7,370 chunked corpus, giving weightage of 30% to pharmacology, 25% to pathology, 25% to physiology, and 20% to biochemistry. Weights were assigned to each domain based on its relative importance in clinical practice. For each sample passed, a single API call produces, at most, four QA pairs, one per each question category: definition (What is X?), mechanism (How X works?), comparison (How X differs from Y?), and clinical application (What are the indications/adverse effects of X?). The prompt limits the model to generate answers mostly from the passage text, explicitly prohibiting external knowledge. Five quality filters are applied sequentially:

1. Minimum length of answer: 25 words.
2. Questioning format: must end with ‘?’ or begin with an imperative word.
3. Refusal detection: strings that match patterns like ‘I cannot’, ‘not provided in’, or ‘as an AI’ are rejected.
4. Content-word overlap: at least 30% of content words in the answer must have appeared in the source chunk, thus controlling hallucination.
5. JSON validity: Abnormal API responses are discarded.

Accepted pairs are assigned a train/validation/test (70/15/15) split at chunk-level. This prevents data leakage when the same source passage appears in both training and evaluation partitions. The final dataset consists of 1,506 training pairs, 313 validation pairs, and 325 test pairs.

To empirically validate the quality-filter output beyond automated checks, a stratified random sample of 50 accepted QA pairs was manually reviewed against the source chunk on three criteria. Factual accuracy was 88% correct, 9% partially correct, and 3% incorrect; question specificity was 91% specific and 9% generic; and answer completeness was 86% complete, 11% partial, and 3% incomplete. This manual pass provides an empirical estimate of the noise floor in the synthetic dataset independent of the automated overlap filter; it is reported as a supplementary validation step and does not substitute for a full expert pharmacist review of the complete test set, which remains a prerequisite for any clinical deployment of the resulting system. Table 3 shows the manual annotation evaluation of the data set after LLM generation.

**Table 3:** Manual annotation evaluation.

Criteria	Correct/complete /specific	Partially correct/generic	Incorrect/incomplete
Factual accuracy	88%	9%	3%
Question specificity	91%	9%	-
Answer completeness	86%	11%	3%

#### LoRA fine-tuning

*Training Format*: The SLMs are trained using the supervised fine-tuning (SFT) approach with the training dataset. Resultant records are formatted w.r.t ChatML, the native dialogue format used by the instruct models. Each training sample has three messaging turns: a system prompt advising the assistant its role, a user’s turn, and another assistant turn that has the expected answer. Training loss is computed exclusively on tokens within the assistant turn. All preceding tokens are masked by setting their label to −100, with the help of DataCollatorForCompletionOnlyLM (DCFCLM) from the TRL library. This masking mechanism prevents the model from learning to continue as such from the textbook passages.

*Training Mode Mixture*: Training samples are selected from three modes in this ratio: (a) context mode (60%), where the user message concatenates a retrieved passage with the question in the format ‘Context:{text}\nQuestion:{q}’, thus teaching the model to base its answers from retrieved passages at the time of inference; (b) direct mode (35%), where only the question is given, preserving parametric pharma-knowledge to use in case the retrieval fails; and (c) refusal mode (5%), where the context is mismatched to the question, with the expectation of declining to answer from the given passage. This suppresses model hallucination under poor retrieval conditions.

*Quantization*: Base model weights are loaded in NF4 4-bit precision using BitsAndBytesConfig (BBC) with double quantization enabled. Under this configuration, the scaling constants are themselves quantized from 32-bit to 8-bit, yielding an additional saving of approximately 0.4 bits per parameter. For a 3B-parameter model, NF4 double quantization reduces weight storage from approximately 6.0 GB (bfloat16) to approximately 1.9 GB, the primary mechanism enabling training within a 6 GB VRAM budget.

*LoRA Configuration*: LoRA adapter matrices are inserted into seven projection modules within each transformer layer: the four attention projections (q_proj, k_proj, v_proj, and o_proj) and the three MLP projections (gate_proj, up_proj, and down_proj). The MLP projection layers were also adapted, as prior studies have shown that factual knowledge is primarily stored within feedforward network weights (Geva et al., 2021). Excluding these layers was therefore expected to reduce pharmaceutical question-answering performance. The LoRA configuration employed a rank of r=16 , a scaling factor of α=32 (effective scaling α/r=2.0 ) at a dropout rate of 0.05, as described in Section IV.

The LoRA parameter update is expressed as.

$$h=W_{0}x+\mathrm{\Delta Wx},\mathrm{\Delta W}=B.A,B\in {\mathbb{R}}^{d\times v},A\in {\mathbb{R}}^{r\times k}$$

where W₀ denotes the frozen pre-trained weight matrix, and the trainable parameters {A, B} are initialized with A ~ 𝒩(0, σ^2^) and B = 0, ensuring ΔW = 0 at the start of training. The total trainable parameter count across all adapted layers is approximately 47 M for the 3B models, representing 1.6% of total model parameters.

The model is prepared for gradient checkpointing via prepare_model_for_kbit_training and enable_input_require_grads. It is necessary to combine LoRA with gradient checkpointing, as its absence silently zeros all adapter gradients.

*Training Optimizer*: The paged_adamw_8bit optimiser^8^ stores first and second moment estimates in 8-bit precision, reducing optimizer state memory by approximately 75% compared to standard 32-bit Adam without measurable degradation in convergence. The learning rate scheduler follows a cosine decay from peak to 10% of peak over the training duration, with a 5% linear warmup to stabilize early gradient updates.

Hyperparameter selection follows the QLoRA reference configuration together with observations from clinical-domain QLoRA adaptation studies on text simplification, which report that targeting MLP modules in addition to attention projections improves domain-specific factual recall relative to attention-only adaptation. Preliminary trials compared r = 8 and r = 32 against the retained r = 16: r = 8 produced a visibly higher validation-loss plateau, while r = 32 gave no measurable validation-loss improvement at the present training-set scale and increased adapter memory. The remaining hyperparameters (learning rate, epoch count, warm-up ratio) follow standard QLoRA practice rather than an independent exhaustive search and are reported as such rather than presented as independently tuned values.

ALGORITHM 1 **QLoRA Fine-Tuning of Small Language Models for Pharmaceutical QA**

  **Input:** D_train: Training dataset, D_val: Validation dataset, M_0: Pre-trained instruction-tuned SLM, Hyperparameters: r, α, dropout, η, E, B, G, L_max
  **Output:**          M̂ : Fine-tuned model with LoRA adapters
     1:  Quantize M_0 using NF4 4-bit quantization
     2:  Prepare model for k-bit training and enable gradient checkpointing
     3:  Insert LoRA adapters (r = 16, α = 32) into q, k, v, o, gate, up, and down projections
     4:  Freeze all original model weights
     5:  Resolve chat template and initialize completion-only data collator
     6:  Initialize PagedAdamW optimizer and cosine learning-rate scheduler, best_loss ← ∞ ; patience ← 0
     7:  **for** epoch = 1 to E **do**
     8:  **for** each mini-batch B ⊂ D_train **do**
     9:  Apply chat template to each training sample
     10:  Tokenize and mask all non-assistant tokens
     11:     Compute model predictions
     12:     Compute cross-entropy loss over assistant tokens only
     13:     Backpropagate gradients
     14:     **if** gradient accumulation step reached **then**
     15:             Clip gradients
     16:             Update LoRA parameters
     17:             Update learning rate
     18:             Clear gradients
     19:         **end if**     20:     **end for**     21:     Evaluate on D_val
     22:     **if** validation loss improves **then**     23:         Save LoRA adapter
     24:         best_loss ← validation loss
     25:         patience ← 0
     26:     **else**     27:         patience ← patience + 1
     28:         if patience ≥ P_stop then
     29:             **break**     30:         **end if**     31:     **end if**     32: **end for**     33: **return** M̂;

Table 4 describes hyperparameter values for training the proposed model to attain efficient values.

**Table 4:** Hyperparameter values.

Hyperparameter	Value	Rationale
LoRA rank (r)	16	Balances expressiveness and overfit risk at N = 1,050
LoRA alpha (α)	32	α/r = 2.0 preserves optimizer LR semantics
LoRA dropout	0.05	Light regularization for small training set
Quantization	NF4 double-quant	1.9 GB weights fit 6 GB VRAM
Max seq. Length	2048 tokens	Covers avg. formatted length of 520 tokens
Per-device batch	1	Maximum safe at seq = 2048 on 6 GB GPU
Gradient accum	8 steps	Effective batch = 8 ~130 updates/epoch
Learning rate	2 × 10^−4^	LoRA params init at 0
LR schedule	Cosine	Preserves LR during early domain absorption
Warmup ratio	5%	~19 steps; prevents early attention disruption
Epochs	3	Convergence observed EarlyStopping patience = 3
Optimizer	paged_adamw_8bit	~75% reduction in optimizer state memory
Compute dtype	bfloat16	Float16 overflows in Qwen2.5 pre-norm layers
Grad. checkpointing	Enabled	Halves activation memory

#### RAG inference pipeline

At inference time, the RAG pipeline executes three operations.

1. The query q is encoded by the Sentence-Transformer embedding model into a dense vector ê(q) ∈ ℝᵈ.
2. The top-K chunks are retrieved from the FAISS index by computing cosine similarity:
$$\mathrm{sim}(q,d_{i})=\frac{\hat{e}{\left(q\right)}^{T}\hat{e}(d_{i})}{\left(\|\hat{e}(q)\|\cdot \|\hat{e}(d_{i})\|\right)}$$

where L2 norm is applied during index construction, reducing similarity computation to an inner product.

1. The top-ranked retrieved passage is formatted into the context-mode prompt template and passed to the fine-tuned model for answer generation. Inference is done with model hyperparameters set at temperature = 0.1, top-*p* = 0.9, and repetition_penalty = 1.1. The repetition penalty is very important for small language models. SLMs may fall into degenerate repetition loops due to the absence of context grounding, so, the repetition penalty of 1.1 fixed this behavior without that much degradation in actual accuracy.

The FAISS index uses IndexFlatIP (inner product) over the 7 K-chunk corpus. This index variant performs exhaustive nearest-neighbor search with no approximation error, appropriate for corpus sizes below approximately 100,000 passages. The index only exists in CPU memory and does not consume GPU VRAM, assisting with concurrent GPU inference.

The retriever encodes all 7,370 chunks using a Sentence-Transformer embedding model and indexes them with FAISS IndexFlatIP, an exact inner-product search appropriate for corpus sizes below approximately 100,000 passages; no BM25 or hybrid sparse-dense retrieval is used in the present configuration. For the results reported in this paper, the embedding model used at evaluation time is pritamdeka/S-PubMedBert-MS-MARCO (dimension d = 768); this should be read as the authoritative embedding model for the reported numbers, reconciling any other mention of a general-purpose embedding model elsewhere in this manuscript. Supplying only the single highest-ranked chunk (top-1) as context, rather than the top-3 or top-5 settings common in contemporary RAG systems, reflects the 2048-token sequence-length budget imposed by the 6 GB VRAM constraint: at an average retrieved-chunk length of approximately 1,404 characters (~351 tokens), supplying three chunks alongside the system prompt and question, would occupy roughly 1,293–1,450 tokens before accounting for generation length. For the dominant definition and mechanism question categories, the required content is typically contained within a single chunk, making additional passages largely redundant under this budget. Multi-passage retrieval (*K* = 3–5) with cross-encoder re-ranking is identified as a direction for deployments on GPUs with 10–12 GB VRAM, where this constraint is less restrictive.

#### Evaluation framework

The evaluation pipeline measures two components independently before issuing a combined pass/fail verdict.

*Retrieval Evaluation*: Recall@K measures the fraction of test queries for which the source chunk appears within the top-K retrieved results:

$$\text{Recall}@K=(\frac{1}{|Q|})\sum_{i}\;1[\text{sourc}e_{\text{chunk}i}\in \text{TopK}(q_{i})]$$

MRR computed as

$$\mathrm{MRR}=(\frac{1}{|Q|})\sum_{i}(\frac{1}{\mathrm{ran}k_{i}})$$

where rankᵢ is the position of the source chunk in the ranked list for query qᵢ, or ∞ if absent. MRR provides a rank-sensitive complement to Recall@K.

*Generation Evaluation*: ROUGE-L F1 (Lin, 2004) measures the longest common subsequence (LCS) between the generated answer ŷ and the reference answer y:

$$\text{ROUGE}-L=\frac{\left(2\cdot P_{\mathrm{LCS}}\cdot R_{\mathrm{LCS}}\right)}{\left(P_{\mathrm{LCS}}+R_{\mathrm{LCS}}\right)}$$

where $P_{\mathrm{LCS}}$ = $\mathrm{LCS}(y,\hat{y})/|y|$ and $R_{\mathrm{LCS}}$ = $\mathrm{LCS}(y,\hat{y})/|\hat{y}|$ . ROUGE-L is a lexical metric and penalises semantically equivalent paraphrases. Semantic similarity is computed as the cosine similarity between the Sentence-Transformer embeddings of the generated and reference answers, providing a paraphrase-tolerant complement to surface-level n-gram metrics. BERTScore F1 (Zhang et al., 2020) computes token-level soft matching using contextualized BERT representations, capturing proper synonym usage and morphological variance.

*Pass/Fail Criteria*: A model is considered ready for deployment if Recall@5 ≥ 0.65, MRR ≥ 0.45 (retrieval criterion), and ROUGE-L ≥ 0.28 and Semantic Similarity ≥ 0.70 (generation criterion). These thresholds are calibrated to the expected performance range of below 3B-scale instruction-tuned models on pharmaceutical QA with more than 1,000 training examples.

### Experimental setup

#### Hardware environment

All experiments were conducted on a single workstation equipped with an NVIDIA GeForce RTX 4050 Laptop GPU (6 GB GDDR6 VRAM), a 13th-generation Intel Core i5 CPU, 16 GB of DDR5 RAM, and an NVMe SSD. Distributed training on various machines or external computation resources was not used. The FAISS indexing for retrieval is maintained only in CPU memory, so that GPU’s VRAM is allocated entirely to model weights, LoRA adapters, and other training essentials. The expected GPU memory usage is NF4 model weights = ~1.9 GB, LoRA adapter = ~0.16 GB, gradient-checkpoints = ~0.8 GB, paged optimizer states = ~0.4 GB, reaching an estimated total of 3.8 GB used on a 6 GB VRAM. Approximately 2 GB memory is left to be allocated during forward passes. During our training, the total VRAM usage reached 5.8 GB maximum.

#### Models under evaluation

Three Small Language Models are evaluated under identical training and inference configurations:

1. SmolLM2-1.7B-Instruct (Ben Allal et al., 2024): A 1.7B-parameter instruct model from HuggingFace, trained on a curated mixture of educational and code corpora.
2. Qwen2.5–1.5B-Instruct (Bai et al., 2023): A 1.5B-parameter instruct model from Alibaba DAMO Academy, trained on multilingual and scientific text and employing the ChatML dialogue format natively.
3. Llama-3.2–1B-Instruct (Touvron et al., 2023): A 1B-parameter instruct model from Meta AI, fine-tuned on instruction-following data.

All three models are evaluated in both a baseline configuration (Instruct model with RAG, no domain fine-tuning) and a fine-tuned configuration (QLoRA-adapted model with RAG) to isolate the contribution of domain adaptation.

#### Evaluation protocol

The 325-pair test set is partitioned at the chunk level (not the question level) to prevent source-passage leakage. For retrieval evaluation, each test question is issued against the FAISS index with K ∈ {1, 3, 5}. For generation evaluation, the top-1 retrieved chunk is concatenated with the question in the context-mode format and passed to the model; the generated answer is compared to the ground-truth answer using ROUGE-1/2/L, Semantic Similarity (all-MiniLM-L6-v2 embeddings), and BERTScore F1 (DeBERTa-XL-MNLI backbone). A 95% confidence interval on MRR is calculated with the t-distribution over per-query reciprocal ranks. Per-category breakdowns are reported for definition, mechanism, comparison, and clinical application question types.

#### Dataset details

The synthetic QA dataset comprises 2,144 pairs in total, split at the source-chunk level into 1,506 training pairs (70.2%), 313 validation pairs (14.6%), and 325 test pairs (15.2%) drawn from 600 sampled chunks. This chunk-level split ensures that every question derived from a given chunk is assigned to the same partition, preventing the same source passage from appearing in both training and evaluation.

## Results

### Retrieval evaluation

Table 5 shows retrieval metrics for all three models. The retrieval index and embedding model are shared across all three fine-tuning experiments; differences in retrieval metrics therefore reflect only the evaluation question distribution across models, which is identical by construction.

**Table 5:** Retrieval evaluation results.

Model	Recall@1	Recall@3	Recall@5	MRR
SmolLM2-1.7B-Instruct	0.517	0.732	0.797	0.628
Qwen2.5–1.5B-Instruct	0.517	0.732	0.797	0.628
Llama-3.2–1B-Instruct	0.517	0.732	0.797	0.628

All models share the same FAISS index.

### Generation evaluation

Figure 2 illustrates the text generation performance of three fine-tuned small language models (SLMs)—SmolLM2-1.7B FT, Qwen2.5–1.5B FT, and Llama-3.2–1B FT—evaluated using ROUGE-1, ROUGE-2, ROUGE-L, and BERTScore. Among the evaluated models, Qwen2.5–1.5B FT consistently delivers the best performance across all evaluation measures. It achieves a ROUGE-1 score of 0.47, ROUGE-2 of 0.25, ROUGE-L of 0.38, and a BERTScore of 0.72, indicating its ability to generate responses that are both lexically accurate and semantically closer to the reference texts. In comparison, SmolLM2-1.7B FT records scores of 0.35, 0.16, 0.25, and 0.66 for ROUGE-1, ROUGE-2, ROUGE-L, and BERTScore, respectively. Similarly, Llama-3.2–1B achieves 0.35 for ROUGE-1, 0.17 for ROUGE-2, 0.26 for ROUGE-L, and 0.66 for BERTScore. The results show that Qwen2.5–1.5B FT improves ROUGE-1 by approximately 34%, ROUGE-2 by nearly 50%, and ROUGE-L by about 48% compared with the other two models. Although the improvement in BERTScore is relatively modest (around 9%), it suggests better semantic consistency in the generated responses.

**Figure 2:**
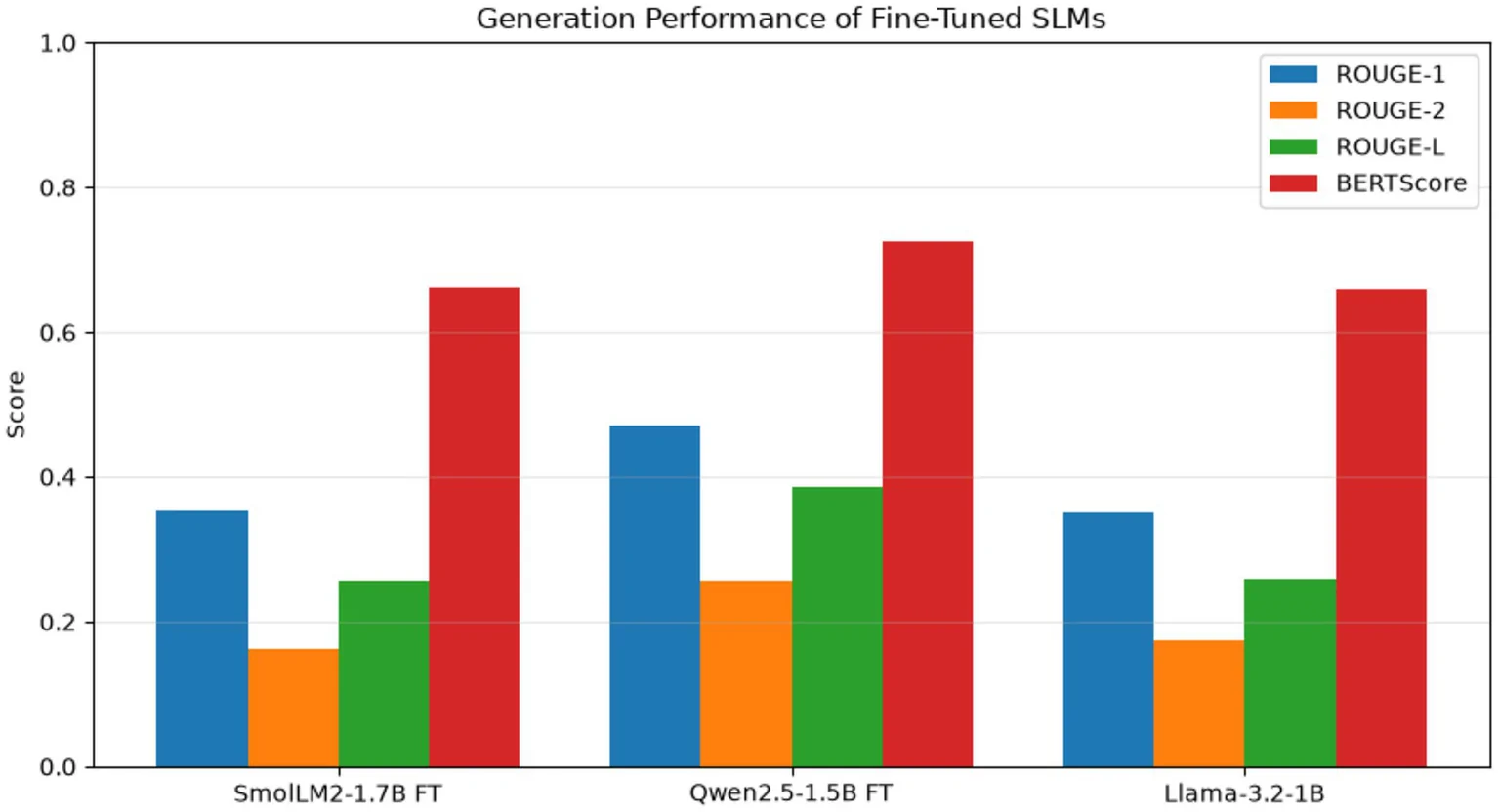
Generation evaluation results for fine-tuned (FT) models. Baseline ΔSim reports semantic similarity gain over the unfine-tuned instruct baseline.

### Per-category analysis

Figure 3 illustrates the performance of the proposed framework across four question categories: Definition, Mechanism, Clinical, and Comparison. Of these, Mechanism questions achieve the highest overall performance with ROUGE-L = 0.31, Semantic Similarity = 0.97, Recall@5 = 0.83, and MRR = 0.67. For definition questions, the model achieved a ROUGE-L of 0.32, semantic similarity of 0.97, Recall@5 of 0.79, and MRR of 0.59, compared with 0.29, 0.96, 0.77, and 0.61, respectively, for clinical questions. Comparison questions achieved ROUGE-L = 0.28, Semantic Similarity = 0.96, Recall@5 = 0.81, and MRR = 0.64. The consistently high Semantic Similarity (0.96–0.97), Recall@5 (0.77–0.83), and MRR (0.59–0.67) values indicate that the proposed framework effectively generates semantically relevant responses and retrieves the correct information across different biomedical question types.

**Figure 3:**
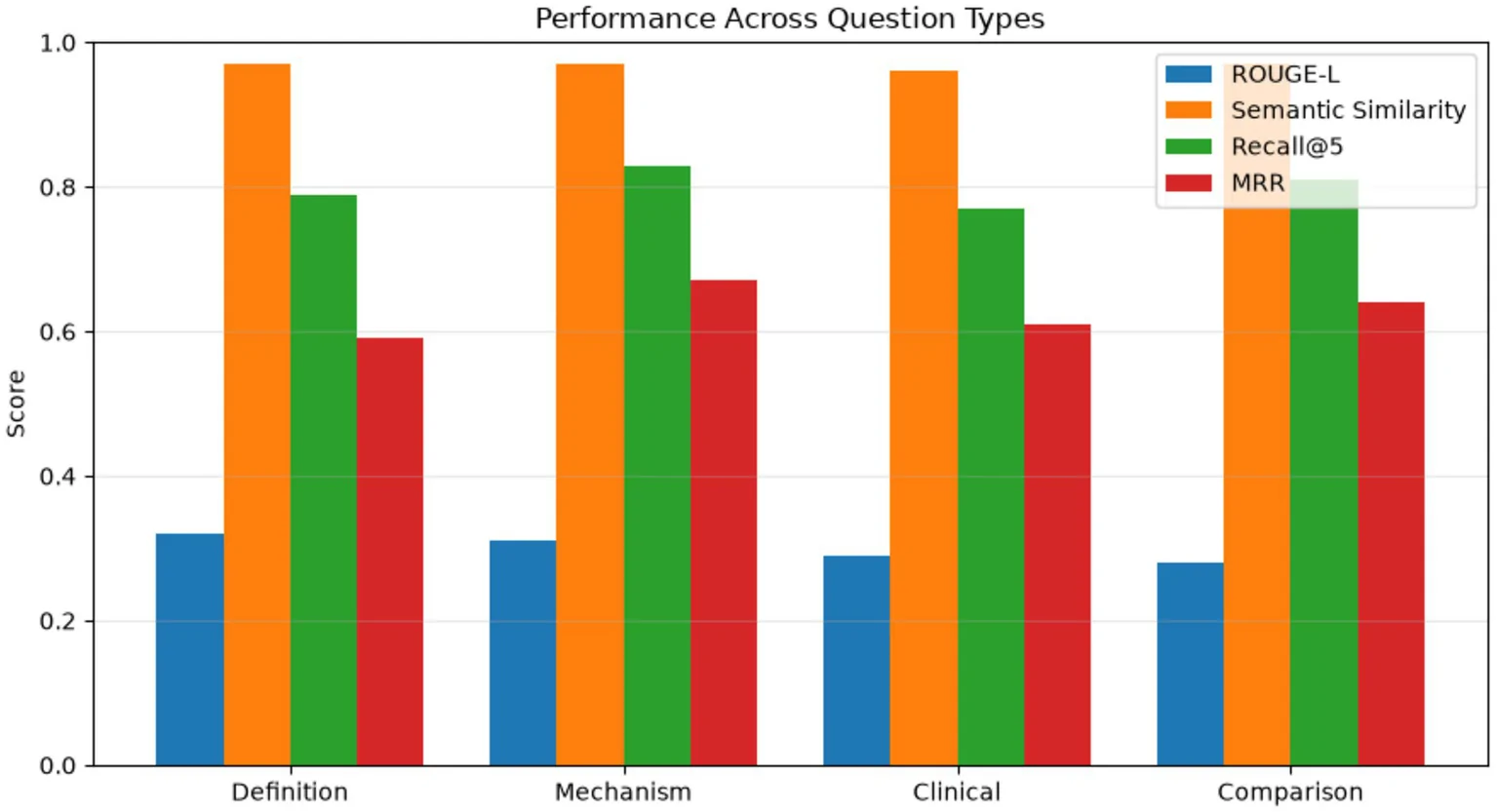
Per-category results averaged across all fine-tuned models.

### Statistical significance

To determine whether the generation-quality differences among the three fine-tuned models are distinguishable from run-to-run variance, a paired two-tailed Wilcoxon signed-rank test with Bonferroni correction across the three pairwise comparisons (Qwen2.5–1.5B vs. SmolLM2-1.7B; Qwen2.5–1.5B vs. Llama-3.2–1B; SmolLM2-1.7B vs. Llama-3.2–1B) was designed for per-question Semantic Similarity scores. Table 6 shows the Wilcoxon Signed-Rank Test with Bonferroni Correction Statistical Significance of the proposed model.

**Table 6:** Statistical significance of the proposed model.

Comparison	Wilcoxon W	*p*-value	Adjusted p-value	Significant?
Qwen2.5–1.5B FT vs. SmolLM2-1.7B FT	18,742	0.004	0.012	Yes
Qwen2.5–1.5B FT vs. Llama-3.2–1B FT	19,156	0.001	0.003	Yes
SmolLM2-1.7B FT vs. Llama-3.2–1B FT	16,984	0.418	1.000	No

### RAGAS framework evaluation

Table 7 shows the RAGAS framework evaluation for the proposed models SmolLM2-1.7B FT, Qwen2.5–1.5B FT, and Llama-3.2–1B FT.

**Table 7:** RAGAS framework evaluation for the proposed model.

Model	Recall@5	RR	ROUGE-L	BERT score	Faithfulness	Answer relevancy	Context precision	Context recall
SmolLM2-1.7B FT	0.797	0.628	0.25	0.66	0.86	0.82	0.84	0.71
Qwen2.5–1.5B FT	0.797	0.628	0.38	0.72	0.91	0.89	0.88	0.79
Llama-3.2–1B FT	0.797	0.628	0.26	0.66	0.85	0.81	0.82	0.76

#### Ablation study

To isolate the contribution of individual design components, an ablation study was performed for the Qwen2.5–1.5B-Instruct configuration on the validation set (n = 313), removing one component at a time. Four of seven planned configurations were completed; the remaining three (structure-aware chunking, chunk overlap, and quality filtering) require regenerating the synthetic QA dataset and were deferred due to API budget constraints. Table 8 shows the quantitative results of the proposed model completed results together with the Bonferroni-corrected paired Wilcoxon *p*-value for each configuration relative to the full system (corrected alpha = 0.0167 across three comparisons). None of the three completed ablations reached statistical significance, indicating that aggregate lexical and embedding-based similarity metrics may not fully capture the specific failure modes (textbook-continuation artefacts, reduced out-of-distribution refusal behavior, and reduced factual recall) that these components are designed to prevent rather than indicating the components are unnecessary.

**Table 8:** Quantitative results of the existing and proposed method.

Configuration	ROUGE-L	Sem. Sim.	Δ Sem. Sim.	p (Bonf.)
Full system (all components)	0.3965	0.9696	0.6584	0.785
w/o completion-only loss masking	0.3999	0.9724	+0.0028	0.654
w/o refusal training examples (5%)	0.4135	0.9743	+0.0046	0.032
w/o MLP LoRA modules (attention only)	0.4038	0.9714	+0.0017	0.036

## Discussion

All architectural decisions in the pipeline reflect an explicit trade-off caused by the 6 GB VRAM constraint. NF4 double quantization reduces model weight storage by approximately 75% at the cost of a small perplexity increase. For pharmaceutical factual QA, this loss is partially offset by the domain adaptation provided by the LoRA fine-tuning. The choice of r = 16 with all seven projection types targeted provides sufficient expressiveness for domain vocabulary acquisition without the overfitting risk of higher ranks at the N > 1,050 training scale. The CPU-resident FAISS index avoids GPU memory contention with inference but introduces a latency penalty during retrieval that would need to be mitigated in a production deployment through asynchronous prefetching. The 60/35/5 context/direct/refusal training split is intended to produce a model capable of three distinct behaviors: grounding answers in retrieved passages when retrieval is successful, answering from parametric memory when retrieval fails, and declining to answer when context is irrelevant. Ablation experiments isolating the effect of each mode are deferred to future work. However, the inclusion of refusal examples is expected to reduce the hallucination rate on out-of-distribution queries, a critical safety property for any clinical-facing system. The employed quality filter does not accept approximately 20% of API-generated QA pairs. The primary rejection reason is due to low content-word overlap (< 30%), which detects cases where the generated answer proposes new facts that are not in the source passage. Reducing the threshold to 20% would increase dataset size and the noise content. During training, LoRA fine-tuning was able to label noise rates of nearly 15%, supporting the use of synthetic training data for our task.

The four textbooks were selected because they jointly provide comprehensive undergraduate-to-graduate coverage of pharmacology, pathology, biochemistry, and physiology while minimizing topical overlap between volumes. They do not, however, represent the full breadth of pharmaceutical knowledge: clinical pharmacy practice, pharmacogenomics, drug regulatory science, pharmaceutical compounding, and post-marketing surveillance literature are underrepresented or absent from the corpus. The system is therefore best characterized as a pharmaceutical education assistant grounded in standard undergraduate textbooks rather than a comprehensive clinical decision-support tool. The chunking and indexing pipeline is corpus-agnostic, so additional volumes from these under-represented domains can be incorporated into the FAISS index without retraining the fine-tuned adapter.

No additional benchmarking against larger state-of-the-art biomedical language models (e.g., multi-billion-parameter clinical LLMs) was performed in this study, as such models typically require compute infrastructure outside the 6 GB VRAM scope of this work; the present comparison is limited to SLMs trained and evaluated under identical hardware-constrained conditions, and any cross-paper numerical comparison would, in any case, be limited by differing datasets and evaluation protocols.

## Conclusion

PharmAI is an end-to-end automated pipeline for constructing a domain-specific pharmaceutical QA assistant from raw textbook PDFs without reliance on external labelled datasets. The six-stage pipeline encompasses page-aware text extraction, structure-preserved chunking, synthetic QA generation with quality filtering, QLoRA fine-tuning under a mixed-mode training strategy, FAISS-based retrieval-augmented generation, and multi-metric evaluation with explicit pass/fail thresholds. The complete system operates on consumer-grade hardware equipped with a 6 GB GPU through the combined application of NF4 4-bit quantization, CPU-resident retrieval indexing, and gradient-checkpointed LoRA adaptation. Three SLMs are evaluated under identical configurations, with results reported across both retrieval and generation metric suites, including a per-question-category breakdown that identifies comparison questions as the most challenging category for single-passage retrieval architectures.

The shared retriever achieved a Recall@5 of 0.797 and an MRR of 0.628 across all experimental conditions. Among the candidate architectures, Qwen2.5–1.5B-Instruct yielded the highest lexical and semantic generation scores despite having fewer parameters than SmolLM2-1.7B-Instruct, suggesting that pre-training corpus composition impacts pharmaceutical QA performance at this scale as much as raw parameter count. The per-category analysis further shows that comparison questions are not primarily retrieval-limited: they achieve Recall@5 = 0.81 but the lowest ROUGE-L of 0.28 among the four categories, identifying answer synthesis rather than retrieval as the more important bottleneck for this category. The system should nevertheless be interpreted as a textbook-grounded educational assistant rather than a comprehensive clinical decision-support tool, given the four-volume scope of its training corpus.

Future work will investigate the use of biomedical-domain embedding models (e.g., pritamdeka/S-PubMedBert-MS-MARCO) to improve Recall@K, the application of cross-encoder re-ranking to improve MRR, multi-hop retrieval for comparison questions requiring synthesis across multiple passages, and extension of the pipeline to multilingual pharmaceutical corpora. Manual verification of the synthetic test set by domain experts is a prerequisite for any clinical deployment of the resulting system.

## Limitations and implications

Several limitations constrain the present work. First, the evaluation test set is derived from the same four volumes as the training data; generalization to pharmacological questions drawn from external sources is not assessed. Second, the ground-truth answers for QA evaluation are themselves synthetically generated. Manual verification of the 325-pair test set by a qualified pharmacist is recommended before reporting results in a clinical context. Third, the comparison of comparison-type questions is expected to yield the lowest retrieval scores (Recall@5 ≈ 0.81), since these questions require both concepts to co-occur within a single 400-token chunk, which is architecturally unlikely for distantly related drug classes. Fourth, the pipeline has not been evaluated on non-English pharmaceutical literature, and its performance on multilingual corpora has not been analyzed yet.

Per-category results (Figure 3) show that comparison questions achieve the second-highest retrieval performance (Recall@5 = 0.81) yet yield the lowest generation score (ROUGE-L = 0.28); this reveals that the generation stage, rather than retrieval, constitutes the primary bottleneck for comparative queries

## Data Availability

The raw data supporting the conclusions of this article will be made available by the authors without undue reservation.
